# Identifying the necessary capacities for the adaptation of a diabetes phenotyping algorithm in countries of differing economic development status

**DOI:** 10.1080/16549716.2022.2157542

**Published:** 2023-01-24

**Authors:** Angela Jackson-Morris, Rita Sembajwe, Feisul Idzwan Mustapha, Arunah Chandran, Simon Pierre Niyonsenga, Crispin Gishoma, Elizabeth Onyango, Zachariah Muriuki, Kavita Dharamraj, Nathan Ellermeier, Rachel Nugent, Rasa Kazlauskaite

**Affiliations:** a Center for Global Noncommunicable Diseases (AJM, RN, NE), RS Social, Statistical, and Environmental Sciences, RTI International, Seattle, WA, USA; b NCD section, Disease Control Division, Malaysia Ministry of Health, Kuala Lumpur, Malaysia; c Department of Biotechnology, Rwanda Biomedical Centre, Kigali, Rwanda; d Rwanda Diabetes Association, Kigali, Rwanda; e Division of Non-Communicable Disease Prevention and Control, Kenya Ministry of Health, Nairobi, Kenya; f South Western Regional Health Authority, Princes Town, Trinidad and Tobago; g Center for Global Noncommunicable Diseases, Consultant, Durham, NC, USA; h Department of Internal Medicine, RUSH University Medical Center, Chicago, IL, USA

**Keywords:** Diabetes, health information system, non-communicable diseases, LMICs, phenotype

## Abstract

**Background:**

In 2019, the World Health Organization recognised diabetes as a clinically and pathophysiologically heterogeneous set of related diseases. Little is currently known about the diabetes phenotypes in the population of low- and middle-income countries (LMICs), yet identifying their different risks and aetiology has great potential to guide the development of more effective, tailored prevention and treatment.

**Objectives:**

This study reviewed the scope of diabetes datasets, health information ecosystems, and human resource capacity in four countries to assess whether a diabetes phenotyping algorithm (developed under a companion study) could be successfully applied.

**Methods:**

The capacity assessment was undertaken with four countries: Trinidad, Malaysia, Kenya, and Rwanda. Diabetes programme staff completed a checklist of available diabetes data variables and then participated in semi-structured interviews about Health Information System (HIS) ecosystem conditions, diabetes programme context, and human resource needs. Descriptive analysis was undertaken.

**Results:**

Only Malaysia collected the full set of the required diabetes data for the diabetes algorithm, although all countries did collect the required diabetes complication data. An HIS ecosystem existed in all settings, with variations in data hosting and sharing. All countries had access to HIS or ICT support, and epidemiologists or biostatisticians to support dataset preparation and algorithm application.

**Conclusions:**

Malaysia was found to be most ready to apply the phenotyping algorithm. A fundamental impediment in the other settings was the absence of several core diabetes data variables. Additionally, if countries digitise diabetes data collection and centralise diabetes data hosting, this will simplify dataset preparation for algorithm application. These issues reflect common LMIC health systems’ weaknesses in relation to diabetes care, and specifically highlight the importance of investment in improving diabetes data, which can guide population-tailored prevention and management approaches.

## Introduction

Diabetes increased by an extraordinary 70% between 2000 and 2016 to become the ninth largest cause of death globally [1]. Prevalence has risen most rapidly in low- and middle-income countries (LMICs), where the percentage of premature deaths attributable to high blood glucose or diabetes is also higher – with the mortality rate in lower-middle-income countries increasing by 10% more than in high-income countries (HICs) between 2000 and 2019 [2]. The slow and limited adaptations of LMIC health systems to deal with the escalating prevalence of noncommunicable diseases (NCDs) underly this differential. Diabetes risk factors are increasing globally; however, diagnosis, screening, treatment, and disease management are unavailable or unaffordable in many LMICs [3].

Access to data is a weakness of LMIC health systems. In many LMICs, data from patients living with diabetes are only sporadically recorded by electronic patient management systems (e.g. electronic health records, patient registers) at patient visits. Diabetes data that are collected are rarely hosted in a centralised disease registry or shared with national health management information systems (HMIS). These inadequate diabetes data collection, disparate hosting, and uncoordinated data sharing impede an accurate view of a country’s diabetes burden. This leads to poor planning and resource allocation to diabetes programmes – such as an inability to quantify demand for insulin and diabetes supplies – and results in poor quality care for diabetes patients, as well as exacerbating common comorbidities such as HIV and tuberculosis [4].

Moreover, diabetes data are critical to help health professionals classify the condition and select appropriate treatment and secondary prevention. In 2019, the World Health Organisation (WHO) identified the need to reclassify diabetes from a century-old, two-type model to better reflect the clinical and pathophysiological heterogeneity of a multiplicity of related diseases with different aetiology, comorbid risks, and risk of complications. Developing a better understanding of phenotypes was identified as an important element of providing a more effective primary and secondary prevention and management [5]. Identifying more specific phenotypes is particularly important for LMICs as the two-type model was developed using HIC population evidence and has been critiqued as inadequately reflecting differences in risks, disease aetiology, and risk of complications in LMICs [6]. Understanding the specific phenotypes within LMIC populations can guide evidence-based decision-making and appropriate resource allocation for disease management, prevention, and service planning – including options to integrate care with other issues [7].

**Figure 1.**
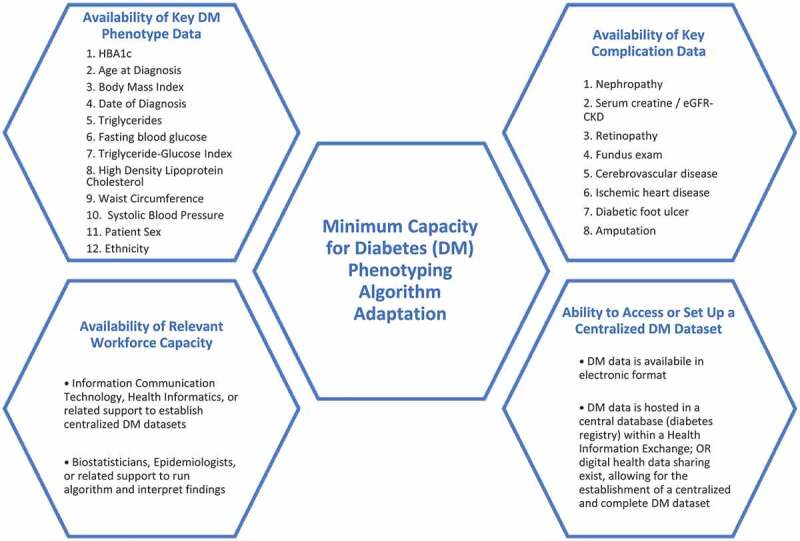
Essential capacities for diabetes algorithm adaptation.

A study to develop and test a prototype algorithm to identify phenotypes was conducted by RUSH University, RTI International, and the Malaysian Ministry of Health [8]. This algorithm requires data relating to diabetes characteristics, outcomes, and patient socio-demographics. Such patient details can inform more customised clinical care and improve patient outcomes. The data must be in a centralised format, such as an electronic registry, which can be digitally mined by skilled health informaticians or biostatisticians. This paper presents a companion study, which assessed the potential for the algorithm to be applied in countries with different economic status and differential capacities, particularly to collect diabetes and complication data; to centrally host and share data in a standardised manner; and availability of human resources to establish a digital diabetes dataset, apply the phenotype algorithm, and analyse and interpret results (Figure 1).

### Objective

The aim of this study is to understand the extent to which countries with varying capacities may successfully adapt and apply phenotyping algorithms, to identify the minimum requirements, and to highlight gaps that impede algorithm implementation.

## Methods

### Subjects

Diabetes programmes in four countries with varying economic status were assessed for their ability to meet those criteria: Trinidad (high income), Malaysia (upper middle income), Kenya (lower middle income), and Rwanda (low income). We sought to assess diabetes programmes in settings with varying resource availability, and where this may impact diabetes programming and health information system (HIS) ecosystem maturity (particularly, data sharing and hosting). The assessed programmes included the following: in Trinidad, a regional programme under the South-West Regional Health Authority, which monitors Type 1 and 2 diabetes through health facility data; in Malaysia, a national programme monitoring Type 1 and 2 diabetes via the National Diabetes Registry at public primary care clinics; in Kenya, a national programme, across a decentralised health system, monitoring Type 1 and 2 diabetes via health facility patient registers and electronic health records, which report to the national HMIS; and in Rwanda, a national programme, under Rwanda Biomedical Centre (RBC), monitoring Type 1 and Type 2 diabetes through its health facilities and national registry (including Type 1 data from Rwanda Diabetes Association facilities).

### Data collection

Prior to their interview, the diabetes programme representatives completed a pre-assessment checklist. This checklist identified the presence or absence of data variables required for phenotyping analysis (clinical indicators and complications). The pre-assessment gave programmes opportunity to investigate and answer questions about their diabetes data in advance of the interview and guided the assessment team’s approach.

The interview questions were related to national diabetes prevalence, and diabetes programme aims and stage of development in order to understand country’s context and priorities and the potential importance of undertaking a phenotyping analysis. Additional topics included how the country reports, hosts, and shares data across HIS ecosystem [9]; the health data completeness and accessibility; access to information communication technology (ICT); health informatics human resources; and access to biostatisticians or epidemiologists for data analysis.

Virtual interviews were held with one to two management-level diabetes programme representatives in each country. Email correspondence followed for clarifications.

### Analysis

A descriptive analysis was undertaken to interpret the capacity assessment findings in relation to the minimum requirements to successfully apply the diabetes phenotype algorithm (Figure 1). The major themes included the extent to which diabetes is prioritised as a disease of significance in the country; the presence of essential diabetes and complication data; the presence of diabetes data in electronic format, and the ability or potential for a country to host health data – specifically diabetes data – in a centralised digital dataset within HIS ecosystem; and the ability of a diabetes programme to access human resources in the areas of ICT, health informatics, biostatistics, and epidemiology to support the establishment of a diabetes dataset to run the phenotype algorithm. Each programme’s readiness to adapt and apply the algorithm was assessed based on the extent to which the essential capacities were present.

## Results

### Availability of essential diabetes and complication data

Table 1 indicates the availability of the essential diabetes data variables across the four programmes. Twelve diabetes data variables are essential to analyse diabetes phenotypes using the algorithm.

**Table 1.** Availability of diabetes phenotype data.

	Present in Country Database			
Diabetes Phenotype Minimum Dataset	Kenya	Rwanda	South-West Region of Trinidad	Malaysia
1. HBA1c	☒	☒	☒	☒
2. Age at diagnosis	☒	☒	☒	☒
3. BMI (or more simply, height and weight)	☒	☒	☒	☒
4. Date of diagnosis (disease duration)	☒	☒	☐	☒
5. Triglycerides	☐	☒	☐	☒
6. Fasting blood glucose	☒	☒	☐	☒
7. HDL cholesterol	☐	☒	☐	☒
8. Waist circumference	☒	☒	☒	☒
9. Systolic BP	☒	☒	☒	☒
10. Patient sex	☒	☒	☒	☒
11. Ethnicity	☐	☐	☒	☒
12. TG-Glucose Index (calculated using triglycerides and fasting blood glucose)	☒	☐	☐	☒
Overall phenotype variable availability	75%	83%	58%	100%

Only Malaysia currently collects data for all 12 required variables. In Rwanda, 83% (10 of 12) were available, and in Kenya, 75% (9 of 12). For South-West Trinidad, only 58% (7 of 12) were available. All programmes collected data on the required eight diabetes complication variables. Programmes with the greatest range of diabetes variables reflected the availability of a wider range of tests being undertaken and recorded in HIS. This is the case in Malaysia where diabetes care has been integrated into primary care for some years. Rwanda’s diabetes data availability was slightly greater than in Kenya and may reflect Rwanda’s more advanced surveillance system in which EMR data link into the national HIS. Kenya has made some progress in integrating diabetes into other health services; however, further surveillance and testing development is needed.

In all countries, the eight essential diabetes complication data variables were collected (Table 2).

**Table 2.** Availability of diabetes complication data.

	Collected in Country			
Diabetes Complication Data	Kenya	Rwanda	South-West Region of Trinidad	Malaysia
1. Nephropathy	☒	☒	☒	☒
2. Serum creatinine/eGFR-CKD (continuous measurement)	☒	☒	☒	☒
3. Retinopathy	☒	☒	☒	☒
4. Fundus exam	☒	☒	☒	☒
5. Cerebrovascular disease	☒	☒	☒	☒
6. Ischemic heart disease	☒	☒	☒	☒
7. Diabetic foot ulcer	☒	☒	☒	☒
8. Amputation	☒	☒	☒	☒
Complications data availability	100%	100%	100%	100%

### Ability to access centralised and standardised digital diabetes data

The format of diabetes data is important to determine the level of data source merging, digitisation, and centralisation (i.e. database development) that needs to be achieved before a phenotyping algorithm can be applied. Centralisation of standardised health data, such as in a digital diabetes registry, reduces the amount of preparation needed for algorithm application.

Table 3 indicates that all four diabetes programmes share data across their HIS ecosystems, a practice that has been catalysed by the need to share aggregate health data for routine monitoring, planning, and resource allocation. For this reason, we concluded that at least foundational electronic data exchange infrastructure and practices are present. Nonetheless, in most of the settings, electronic data sharing is not fully occurring between the points of data collection and diabetes registries or national HMIS. Additionally, in some cases, diabetes data are collected by disparate sources, in differing formats (i.e. paper based, digital, or a hybrid).

**Table 3.** Data collection, format, and sharing capacity.

	Kenya	Rwanda	Trinidad (Regional)	Malaysia
Diabetes data formats and data sharing practices				
1. Diabetes (DM) is a priority disease for the country, as indicated by its NCD programme strategy	Yes	Yes	Yes	Yes
2. Country routinely collects Type 1 and/or Type 2 DM data	Type 1 and 2	Type 1 and 2	Type 1 and 2	Type 1 and 2
3. Country routinely collects the DM phenotyping variables (clinical diabetes measures) dataset	Partial set	Partial set	Partial set	Complete set
4. Country routinely collects DM complications data	Yes	Yes	Yes	Yes
Diabetes data formats and data sharing practices				
1. DM reporting/data collection format (electronic, paper, or both)	Paper-based patient registers & EHR	EHR	Paper-based patient registers, tally sheets	Web-based registry
2. DM registry exists electronic, paper, or both	None	Electronic	Microsoft Excel Spreadsheet	Electronic
3. DM data sharing between diabetes case data source and national HMIS occurs (automatic electronic exchange or manual data entry)	Manual data entry	Manual data entry	Manual data entry	N/A
4. Health Information Systems framework exists and is operational in some capacity	Yes	Yes	Yes	Yes

In Rwanda, all data in the diabetes registry come from Electronic Health Record systems (EHRs); however, data have to be manually transferred from independent health-facility-based EHRs into the national registry at the Rwanda Biomedical Centre (RBC). Additionally, diabetes data are routinely submitted to the national HMIS. The existence of a national facility-based-EHRs that collect diabetes data and a national electronic diabetes registry that receives and hosts all Type 2 diabetes data in a centralised and standardised way would require little data preparation prior to algorithm application. In the long term, a higher-quality dataset could be ensured by optimising Rwanda’s HIS infrastructure to enable automatic electronic sharing of diabetes data from health facilities to the RBC Type 2 diabetes registry (via HIE).

Malaysian diabetes data collection occurs through its web-based Type 2 diabetes registry, which is beneficial in potentially requiring less diabetes data preparation before applying the algorithm. However, the registry does not yet share data electronically with the national HMIS. In the long term, expanding the registry to health facilities could create a more representative diabetes dataset upon which to run the algorithm.

In Kenya, diabetes data are collected by several EHR platforms operating at health facilities across the country as well as individual paper-based registers. Aggregate diabetes data are manually entered into the national HMIS at county level. The combination of electronic and paper-based data collection, and the absence of a centralised diabetes registry mean that while diabetes data could be extracted from these disparate sources, it would entail an onerous data conversion process and dataset merging. In the long term, to establish a higher-quality, more complete, diabetes dataset, it would be more efficient to establish a diabetes registry that is interoperable with health facility EHRs (and expanding existing EHRs to replace paper-based patient registers).

Trinidad’s South-West Regional Health Authority (SWRHA) performs paper-based data collection at health facilities, collating data into a Microsoft Excel spreadsheet and using this to report data to a national level. The current SWRHA process provides access to centralised diabetes data, however, in a format more likely to have data quality challenges and reformatting the diabetes data would need to precede algorithm application. In the long term, it would be more efficient to establish an electronic diabetes registry that provides data to the national HMIS.

### Workforce capacity to support algorithm adaptation, data analysis, and interpretation

Table 4 shows that all four diabetes programmes have access to some form of ICT, health informatics, and biostatistics or epidemiology expertise required to support adaptation of diabetes data to digital format and establish data hosting repository and interoperability with electronic systems that supply diabetes data to the repository. Malaysia and Kenya’s diabetes programmes rely on ICT resources shared across all programmes within their Health Ministries. Rwanda’s RBC-based diabetes programme relies on ICT resources shared across RBC programmes. Trinidad SWRHA has regional-level IT support personnel and access to national-level ICT staff. In all four settings, programmes have some access to health informaticians who could potentially support the integration of the algorithm, and data extraction and interpretation. All four diabetes programmes had one or more epidemiologists and/or biostatisticians to lead the interpretation of the findings. Table 5 indicates the readiness of the different programmes to apply the diabetes phenotying algorithm.

**Table 4.** Workforce capacity.

Workforce Capacity for Adaptation of Diabetes Phenotype Algorithm	Kenya	Rwanda	Trinidad (Regional)	Malaysia
1. Workforce capacity for algorithm adaptation, use, and analysis				
2. DM programmes have access to information communication technology human resources to support back-end access to databases	Yes	Yes	Yes	Yes
3. DM programmes have access to health information systems human resources to support algorithm adaptation, extraction, and data interpretation	Yes	Yes	Yes	Yes
4. DM programmes have epidemiologists and/or biostatisticians to support interpretation of data with health practitioners	Yes	Yes	Yes	Yes

**Table 5.** Country readiness to apply the diabetes phenotyping algorithm.

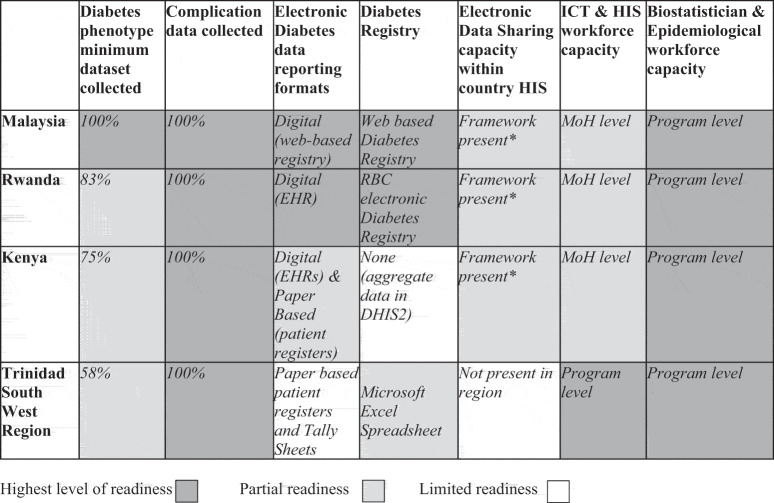

*There are HIE or electronic data sharing frameworks or practices occurring within the health system, but diabetes data are shared manually with national HMIS.

## Discussion

Identifying prevention and treatment measures that are effective and cost-effective is of particular importance in LMIC health systems that are grappling with how to simultaneously address the rapid growth and demands of NCDs, such as diabetes, alongside their existing infectious disease burden [10]. Diabetes phenotyping offers the means to enable a targeted approach to address the different pathophysiological characteristics and clinical needs within the heterogeneous populations of people diagnosed with ‘Type 2 diabetes’ [5,8]. Besides this practical imperative, it is also the case that the two-type classification was originally developed from research in HICs, in an era when diabetes was less prevalent in LMICs [11]. We are now in very different times, when diabetes affects large populations in countries where contextual factors may differ substantially from those in HICs, despite globalising processes. It is also a time of decolonising global health and recognising that LMICs should lead in determining their health futures and responding to population needs [12].

Nonetheless, in order for the phenotyping algorithm to be a practicable strategy for countries, it is essential to first assess whether they currently have the requisite capacities to apply the approach, and if not, to consider how gaps may be addressed. Only one of the four countries assessed by this study was ready to apply the phenotyping algorithm, whereas the fundamental impediment in the other three was the absence of one or more core diabetes data variables. The capacities assessed for this specific purpose represent a sub-set of the total capacities required for effective diabetes care. Nonetheless, they are inextricably linked to the strength and completeness of a country’s diabetes care system, and its strengths and weaknesses. The availability of diabetes variables for phenotyping depends upon diagnostic and testing capacities, which in turn rely upon human resource availability and workforce skillset, medical supplies and equipment. In turn, these facets of diabetes care depend on whether the issue receives sufficient priority and is appropriately resourced within national, regional, and facility delivery plans.

Having data available in a format that allows it to be shared across systems is also essential to be able to apply the phenotyping algorithm. Some countries may have a wealth of data in paper format or in separate electronic systems, yet the value of this for improving prevention and care will not be realised as the data is isolated and cannot easily be extracted, shared, and used within other platforms. Malaysia’s favourable position in this respect was helped by the country’s early investment in diabetes care – which included establishing a digital registry over two decades ago, while Rwanda’s electronic diabetes registry would also enable algorithm application (although the data quality may be less robust due to manual entry from EHR). Whereas Rwanda and Kenya have developed national health information systems in recent decades under the impetus of infectious disease investment – particularly for HIV, these do not yet fully encompass NCDs and therefore the task of obtaining robust diabetes data remains impeded. In Trinidad, the benefits of the SWRHA regional initiative would be magnified if it was supported to develop a digital registry and linked to the national HMIS. Extending the benefits of national HIS to enable digitised, sharable NCD data would greatly advance the understanding of NCDs, including diabetes.

Many LMIC health systems do not yet have strong diabetes care capacities, while at the same time, they face an exponential increase in diabetes prevalence [13]. Consequently, fewer than 1 in 10 people with diabetes in LMICs receive guideline-based comprehensive diabetes treatment [14]. There is therefore a dovetailing of the overall benefits of supporting countries to develop their diabetes care and data management systems, with the capacities required to identify and understand diabetes phenotypes within their populations and particular patient groups. WHO launched a Global Diabetes Compact in 2020 to support countries to implement effective diabetes programmes, with emphasis upon the integration of diabetes prevention, diagnosis, and treatment into primary care [3]. Linking the potential role and requirements for phenotyping into developments under the Compact process has potential to assist and enhance national diabetes care [8].

### Limitations

The study focused on four settings, selected for known diabetes data availability, and to represent countries of varied income/resource status. Other countries and regions may have differing circumstances and capacities. Key informants were selected based on their centrality within, and knowledge of their countries’ diabetes programmes. While informants were asked to consult with colleagues to gather information related to specific technicalities, however, additional information received was limited.

## Conclusion

Identifying the diabetes phenotypes in low- middle-income countries’ populations has the potential to guide the development of more effective, tailored prevention and treatment. This study found that only one of the four diverse countries assessed could readily apply the phenotyping algorithm. The absence of a few critical diabetes data variables was the main impediment in the other settings, although digitised diabetes data collection and centralised diabetes data hosting would also greatly simplify dataset preparation and ease algorithm application. These limitations highlight the importance of investing in improving diabetes data – as an essential tool to improve prevention, care, and outcomes.

## Paper context

Identifying diabetes phenotypes has the potential to enhance diabetes prevention and treatment. A prior study piloted a phenotyping algorithm in a middle-income country with a national diabetes registry. This study assessed the extent to which the algorithm could be implemented in four countries with differing capacities – diabetes datasets, health information ecosystems, and human resources. The four countries’ readiness to apply the algorithm is reported, and the system requirements for any country to apply the algorithm are identified.
